# The Prognosis and Oncological Predictor of Urachal Carcinoma of the Bladder: A Large Scale Multicenter Cohort Study Analyzed 203 Patients With Long Term Follow-Up

**DOI:** 10.3389/fonc.2021.683190

**Published:** 2021-05-31

**Authors:** Young Dong Yu, Young Hwii Ko, Jong Wook Kim, Seung Il Jung, Seok Ho Kang, Jinsung Park, Ho Kyung Seo, Hyung Joon Kim, Byong Chang Jeong, Tae-Hwan Kim, Se Young Choi, Jong Kil Nam, Ja Yoon Ku, Kwan Joong Joo, Won Sik Jang, Young Eun Yoon, Seok Joong Yun, Sung-Hoo Hong, Jong Jin Oh

**Affiliations:** ^1^Department of Urology, College of Medicine, CHA University, Bundang CHA Hospital, Seongnam, South Korea; ^2^Department of Urology, Yeungnam University Hospital, Daegu, South Korea; ^3^Department of Urology, Korea University College of Medicine, Seoul, South Korea; ^4^Department of Urology, Chonnam National University Medical School, Gwangju, South Korea; ^5^Department of Urology, Korea University School of Medicine, Seoul, South Korea; ^6^Department of Urology, Eulji University Hospital, Eulji University School of Medicine, Daejeon, South Korea; ^7^Department of Urology, Center for Urologic Cancer Hospital, Goyang, South Korea; ^8^Department of Urology, Myunggok Medical Research Institute, Konyang University College of Medicine, Daejeon, South Korea; ^9^Department of Urology, Sungkyunkwan University School of Medicine, Samsung Medical Center, Seoul, South Korea; ^10^Department of Urology, Kyungpook National University College of Medicine, Daegu, South Korea; ^11^Department of Urology, Chung-Ang University Hospital, Chung-Ang University College of Medicine, Seoul, South Korea; ^12^Department of Urology, Pusan National University Yangsan Hospital, Pusan, South Korea; ^13^Department of Urology, Pusan National University Hospital, Busan, South Korea; ^14^Department of Urology, Sungkyunkwan University School of Medicine, Kangbuk Samsung Hospital, Seoul, South Korea; ^15^Department of Urology, Severance Hospital, Urological Science Institute, Yonsei University College of Medicine, Seoul, South Korea; ^16^Department of Urology, Hanyang University Hospital, Seoul, South Korea; ^17^Department of Urology, College of Medicine, Chungbuk National University, Chungbuk National University Hospital, Cheongju, South Korea; ^18^Department of Urology, Seoul St Mary’s Hospital, Seoul, South Korea; ^19^Department of Urology, Seoul National University Bundang Hospital, Seongnam, Korea; ^20^Department of Urology, Seoul National University College of Medicine, Seoul, Korea

**Keywords:** urachal carcinoma, bladder, survival rate, surgical margin, lymphovascular invasion

## Abstract

**Aim:**

This study evaluated the prognosis and survival predictors for bladder urachal carcinoma (UC), based on large scale multicenter cohort with long term follow-up database.

**Methods:**

A total 203 patients with bladder UC treated at 19 hospitals were enrolled. Clinical parameters on carcinoma presentation, diagnosis, and therapeutic methods were reviewed for the primary cancer and for all subsequent recurrences. The stage of UC was stratified by Mayo and Sheldon pathological staging system. Oncological outcomes and the possible clinicopathological parameters associated with survival outcomes were investigated.

**Results:**

The mean age of the patients was 54.2 years. Among the total of 203 patients, stages I, II, III, and IV (Mayo stage) were 48 (23.8%), 108 (53.5%), 23 (11.4%), and 23 (11.4%), respectively. Gross hematuria and bladder irritation symptoms were the two most common initial symptoms. The mean follow-up period was 65 months, and 5-year overall survival rates (OS), cancer-specific survival rates (CSS), and recurrence-free survival rates (RFS) were 88.3, 83.1, and 63.9%, respectively. For the patients with Mayo stage ≥III, OS, CSS, and RFS were significantly decreased to 38.0, 35.2, and 28.4%, respectively. The higher pathological stage (Mayo stage ≥III, Sheldon stage ≥IIIc), positive surgical margin (PSM), and positive lymphovascular invasion (PLM) were independent predictors of shorter OS, CSS, and RFS.

**Conclusion:**

The pathological stage, PSM, and PLM were significantly associated with the survival of UC patients, emphasizing an importance of the complete surgical resection of tumor lesion.

## Introduction

During the fetal development, the urachus obliterates and subsequently forms the median umbilical ligament, a 5–10 cm long fibromuscular canal, which extraperitoneally connects between umbilicus and the roof of bladder in the midline. Incomplete closure of the urachus produces urachal remnants such as urachal cyst, or urachal fistula (1–3). Urachal carcinoma (UC) is relatively rare urologic malignancy, which most frequently occurs within the urachal remnant located at the junction between umbilical ligament and dome of the urinary bladder, whereas it can be even found in any location along the midline of the bladder (4). Regarding the incidence of disease entity, recent retrospective study based on the National Cancer Registry in Ireland presented that UC accounts for 0.3% of overall bladder cancer incidence (5). In addition, previous studies have presented that adenocarcinomas, including signet-ring cell carcinoma or mucinous adenocarcinoma, accounts for almost 90% of histological subtypes of UC (6). UC commonly lacks early clinical symptoms and this results in relatively late diagnosis of the disease whereas the carcinoma is already progressed to an advanced stage such as systemic metastasis at the time of beginning therapeutic intervention (7). The treatment methods of UC are different for localized or metastatic disease, but the current main therapy for localized UC is surgery including partial resection or radical resection (8). Many previous studies have reported that UC cases treated with surgical resection had a median survival of 48 months, and no definite difference in survival rates were observed according to different surgical modalities including partial and radical cystectomy (9). The oncological benefits of bilateral pelvic lymphadenectomy in UC is still highly debatable (6, 10). Moreover, regarding metastatic UC, there is no current standard therapy due to the resistant nature of UC to both chemotherapy and radiation therapy (11). Due to the low prevalence of UC, previous literatures regarding clinical characteristics of UC had a relatively small case series with retrospective nature. Furthermore, studies regarding the Asian population with urachal cancer have been rarely reported (4, 11, 12). Thus, the present study is a retrospective multi-center based research, which primarily investigated the clinical characteristics and oncological outcomes of UC patients treated at overall 19 large scale medical institutions in South Korea. Secondarily, we tried to clarify the clinical and therapeutic factors influencing the oncological outcomes of UC cases.

## Methods

### Study Design and Cohorts

The medical records of patients with UC who were treated in 19 large scale institutions in South Korea between 1994 and 2020 were retrospectively reviewed. Before the data collection, research approvals were obtained from the institutional ethics committee of each hospital involved in the current study. Clinical parameters and pathological outcomes of the cohort such as initial cystoscopy findings, computed tomography (CT) findings, laboratory blood test results including squamous cell carcinoma-related antigen (SCC), carbohydrate antigen 19-9 (CA19-9), and carcinoembryonic antigen (CEA) levels, initial symptoms, treatment modalities, tumor stage, pathologic types of carcinoma, and immunohistochemistry markers were analyzed. Regarding immunohistochemistry markers, surgical tissue specimen analyses for overexpression of antigen KI-67, p53, epidermal growth factor receptor (EGFR), as well as cytokeratin-7/-20 (CK-7/CK-20), and KRAS overexpression were performed as potential tumor markers.

### Survival Analysis

During the follow-up period, all patients underwent routine blood and urine tests, cystoscopy, and abdomino-pelvis CT at each outpatient clinic visit. Visiting interval was different at each hospital, but it was between 2 and 3 months. If there was any evidence of suspected recurrence in cystoscopy, urine cytology or abdomino-pelvis CT scans, further imaging studies including brain magnetic resonance imaging (MRI), bone scintigraphy, and positron emission tomography (PET) scans were additionally undertaken. The recurrence-free survival (RFS), cancer-specific survival (CSS), and overall survival (OS) were evaluated for the study cohort. OS was evaluated from the date of initial diagnosis to the date of death from any cause or the date of last follow-up visit. RFS was defined as the time from surgery to the date of first recurrence confirmed by radiological tests or follow-up biopsy. CSS was calculated from the date of initial diagnosis to the date of death by UC.

### Statistical Analysis

Mean and proportion were used to present categorical data. Multivariate Cox proportional hazards regression analyses were performed to evaluate prognostic and independent factors of survival rates. OS, CSS, and RFS were calculated using the Kaplan–Meier survival analyses with log-rank tests. All statistical analyses were undertaken by using the SPSS package version 24.0 (SPSS Inc., Chicago, IL, USA), and p-values less than 0.05 were considered significant.

## Results

### Patient Characteristics

The baseline characteristics of the cohort are presented in **Table 1**. Overall, 203 patients (male: 125 patients, female: 78 patients) treated for UC from 19 different institutions are included in the current study, while the mean follow-up period was 65 months. The mean age was 54.2 years, and most of the patients (202 patients, 99.5%) showed Eastern Cooperative Oncology Group (ECOG) performance status ≤ grade 1. 78 patients (38.4%/mean smoking amount 7.1 pack–years) had smoking history, whereas 33 patients were current smoker. 71.4% of the cohort (145 patients) showed gross hematuria as the cancer-related initial symptom, and suprapubic symptom was the second most common initial symptom (19 patients, 9.4%). Among the cancer related symptoms, omphalitis or mucosuria was not present with any patient, whereas seven patients (3.4%) were asymptomatic. Solid mass (109 patients, 53.7%) and cystic mass (23 patients, 11.3%) were the most common CT findings, whereas only four patients (2.0%) showed cystic mass with calcification in the initial CT scans. According to the initial blood test results, 94.6 and 89.7% patient accompanied elevated CA19-9 and CEA levels, respectively. Regarding therapeutic modalities, 136 patients (67.0%) were treated with surgery alone, whereas 66 patients (32.5%) underwent adjuvant therapy after surgery. Among the modalities of adjuvant therapy, chemotherapy, radiotherapy, and chemotherapy with radiotherapy were performed to 64 patients (97.0%), one patient (1.5%), and one patient (1.5%), respectively. Only one patient received radiotherapy for neoadjuvant therapy before surgical treatment. Regarding mass excision method, 82.8 and 11.3% of the patients underwent partial cystectomy and radical cystectomy, respectively. The other 5.9% patients had initial transurethral resection of bladder (TUR-B) and subsequent partial cystectomy. *En-bloc* resection of the umbilicus and the median umbilical ligament was performed to 12 patients (5.9%). For adjuvant chemotherapy, cisplatin/5-fluorouracil regimen (22 patients/34.4%) and gemcitabine/cisplatin regimen (14 patients/21.9%) were the most commonly used chemotherapy regimen, whereas the other regimens were applied with similar frequencies (1.5–9.4%).

**Table 1:** Baseline characteristics, n = 203.

Parameters	Values	Parameters	Values
Age, mean ± SD, years	54.2 ± 1.0	Cystic mass with calcification	4 (2.0)
Gender: Female, n (%)	78 (38.4)	Mixed solid and cystic mass with calcification	10 (4.9)
Male, n (%)	125 (61.6)	Treatment modality, n (%)	
BMI, mean ± SD, kg/m^2^	24.1 ± 0.1	Conservative	0 (0.0)
Diabetes mellitus, n (%)	30 (14.8)	Surgery alone	136 (67.0)
Hypertension, n (%)	50 (24.6)	Neoadjuvant therapy + surgery	1 (0.5)
ECOG performance status, n (%)		Surgery + adjuvant therapy	66 (32.5)
Grade 0	169 (83.3)	Chemotherapy alone	0 (0.0)
Grade 1	33 (16.3)	Radiotherapy alone	0 (0.0)
Grade 2	1 (0.5)	Chemotherapy+Radiotherapy	0 (0.0)
Grade 3	0 (0.0)	Neoadjuvant treatment, n (%)	
Grade 4	0 (0.0)	Chemotherapy	0 (0.0)
Grade 5	0 (0.0)	Radiotherapy	1 (100.0)
Family history of urachal or bladder cancer, n (%)	50 (24.6)	Adjuvant treatment, n (%)	
Smoking history, n (%)		Chemotherapy	64 (97.0)
Never smoked	78 (38.4)	Radiotherapy	1 (1.5)
Ex-smoker	45 (22.2)	Chemotherapy+Radiotherapy	1 (1.5)
Current smoker	80 (39.4)	Surgical method, n (%)	
Mean smoking amount, pack year	7.1	Open	133 (65.5)
Initial cystoscopy, n (%)		Laparoscopic	40 (19.7)
Mass lesion observed	185 (91.1)	Robotic	30 (14.8)
Normal findings	18 (8.9)	Mass excision method, n (%)	
Initial LDH level, n (%)		Partial cystectomy	168 (82.8)
<Normal range	50 (24.6)	Radical cystectomy	23 (11.3)
Normal range	13 (6.4)	TUR-B + partial cystectomy	12 (5.9)
>Normal range	140 (69.0)	Umbilectomy + median umbilical ligament resection, n (%)	
Initial SCC antigen elevation, n (%)		Not performed	191 (94.1)
<Normal range	3 (1.5)	Performed	12 (5.9)
Normal range	197 (97.0)	PLND, n (%)	
>Normal range	3 (1.5)	Not performed	156 (76.8)
Initial CA19-9 elevation, n (%)		Performed	47 (23.2)
Normal range	11 (5.4)	Mean preoperational hematologic factors	
>Normal range	192 (94.6)	WBC, cells/μ	6555
Initial CEA elevation, n (%)		Hemoglobin, g/d	13.5
Normal range	21 (10.3)	Platelet, platelets ×10^3^/μ	215
>Normal range	182 (89.7)	Serum calcium, mg/d	8.9
Initial symptoms, n (%)		CRP, mg/L	0.7
Gross hematuria	145 (71.4)	Serum LDH, U/L	269.8
Bladder irritation symptoms (suprapubic pain)	19 (9.4)	Serum uric acid, mg/d	5.4
Urinary tract infection	4 (2.0)	Serum creatinine, mg/d	0.9
Voiding difficulties	12 (5.9)	Adjuvant chemotherapy regimen, n (%)	
Palpable infraumbilical mass	8 (3.9)	None	138 (68.0)
Mucosuria	0 (0.0)	Cisplatin + paclitaxel + ifosfamide	1 (0.5)
Umbilical discharge	1 (0.5)	5-Fluorouracil + doxorubicin + etoposide	3 (1.5)
Omphalitis	0 (0.0)	5-Fluorouracil + doxorubicin + mitomycin	3 (1.5)
Microscopic hematuria	7 (3.4)	Methotrexate + 5-Fluorouracil + epirubicin + cisplatin	4 (2.0)
Asymptomatic—incidental finding	7 (3.4)	Cisplatin + 5-fluorouracil	22 (10.8)
Initial CT findings		MVAC	5 (2.5)
Solid mass	109 (53.7)	GC	14 (6.9)
Calcification	8 (3.9)	FOLFORI	2 (1.0)
Solid mass with calcification	19 (4.2)	FOLFOX	6 (3.0)
Thickened bladder dome	15 (7.4)	5-Fluorouracil + leucovorin	1 (0.5)
Cystic mass	23 (11.3)	EP	2 (1.0)
Mixed solid and cystic mass	15 (7.4)	Gemcit + paclitaxel	2 (1.0)

BMI, body mass index; ECOG, Eastern Cooperative Oncology Group; LDH, lactate dehydrogenase; SCC, Squamous cell carcinoma; CA, Carbohydrate antigen; CEA, Carcinoembryonic antigen; CT, computed tomography; TUR-B, transurethral resection of bladder; PLND, pelvic lymphnode dissection; WBC, white blood cell; MVAC, Methotrexate+vinblastine+doxorubicin+ cisplatin; GC, Gemcitabine+cisplatin; FOLFORI, Leucovorin+5-fluorouracil+irinotecan; FOLFOX, folinic acid+fluorouracil+oxaliplatin; EP, Etoposide+cisplatin.

### Histopathological Outcomes

The histopathological results of the cohort are shown in **Table 2**. More than half of the patients (156 patients/77.3%) were classified as Mayo stage ≤II, and 174 patients (85.7%) were assigned as Sheldon stage ≤IIIB. Mean number of positive regional lymph node invasion at the time of surgery was 0.3 lymph nodes per patient. Positive surgical margin PSM) was confirmed in 17 patients (8.4%), and 48 patients (23.6%) accompanied positive lymphovascular invasion (PLM). Positive urine cytology with malignant cells was observed in only seven patients (3.4%). 21 patients (10.3%) had positive distant metastasis at the time of diagnosis, whereas peritoneum and lung were the two most common metastatic sites. More than half (58.1%) of the patients had tumor size ≥4 cm. Regarding pathologic types of tumor, adenocarcinoma (89.7%) was the most frequent type. The other pathological types include urothelial carcinoma (7.9%), undifferentiated carcinoma (2.0%), and small cell carcinoma (0.5%). For histologic sub-classification, mucinous feature (55.9%) and enteric feature (23.3%) were the two most commonly observed histologic sub-types. Immunohistochemical results presented that elevated expression of EGFR, and p53 was confirmed in 90.1% of the patients, whereas KRAS mutation and increased CK-20 expression were detected in 56.2 and 64.0% of the study population.

**Table 2:** Pathological outcomes of urachal carcinoma patients, n = 203.

Parameters	Values	Parameters	Values
Mayo stage, n (%)		Lymphoepithelioma like features	0 (0.0)
I: Tumor confined to urachus and/or bladder	48 (23.8)	Squamous cell features	7 (3.5)
II: Tumor extending beyond the muscular layer of urachus and/or the bladder	108 (53.5)	Transitional cell/adenomatous features	13 (6.4)
		Clear cell featrues	0 (0.0)
III: Tumor infiltrating the regional lymph node	23 (11.4)	Mucinous features	113 (55.9)
IV: Tumor infiltrating non-regional lymph nodes or other distant sites	23 (11.4)	Non-mucinous features	1 (0.5)
		Urine cytology, n (%)	
Sheldon pathological staging system, n (%)		Negative for malignant cells	163 (80.3)
I: Confined to urachal mucosa	12 (5.9)	Positive for malignant cells	7 (3.4)
II: Invasion confined to urachus	32 (15.8)	Suspicious atypical cells	33 (16.3)
III: Local extension		Positive biomarkers, n (%)	
IIIA: To bladder	92 (45.3)	Ki-67	101 (49.8)
IIIB: Peri-urachal vesical fat	38 (18.7)	p53	183 (90.1)
IIIC: To peritoneum	8 (3.9)	EGFR	163 (80.3)
IIID: To viscera other than bladder	4 (2.0)	^Ŧ^KRAS	114 (56.2)
IV: Metastasis to other organs	17 (8.4)	CK-7	58 (28.6)
Number of lymph nodes removed, mean, (LNs/patient)	3.0	CK-20	130 (64.0)
Number of positive lymph nodes, mean, (LNs/patient)	0.3	Recurrence of urachal cancer, n (%)	82 (40.4)
Positive surgical margin, n (%)	17 (8.4)	Salvage treatment, n (%)	
Positive lymphovascular invasion, n (%)	48 (23.6)	None	121 (59.6)
Positive distant metastasis, n	21	Surgery	14 (6.9)
Metastatic site, n(%): Liver	2 (9.5)	Chemotherapy	3 (1.5)
Lung	4 (19.0)	Radiotherpay	23 (11.3)
Bone	1 (4.8)	Chemotherapy + Radiotherapy	42 (20.7)
Peritoneum	8 (38.1)	OS, mean, months	46.8
Abdominal wall	3 (14.3)	2-year OS, %	98.0
Brain	1 (4.8)	5-year OS, %	88.3
Skin	2 (9.5	5-year OS for Mayo stage ≥III, %	38.0
Tumor size, n (%)		10-year OS, %	69.5
<4 cm	85 (41.9)	CSS, mean, months	44.7
≥4 cm	118 (58.1)	2-year CSS, %	95.9
Pathologic type, n (%)		5-year CSS, %	83.1
Adenocarcinoma	182 (89.7)	5-year CSS for Mayo stage ≥III, %	35.2
Urothelial cancer	16 (7.9)	10-year CSS, %	68.4
Undifferentiated cancer	4 (2.0)	RFS, mean, months	39.6
Small cell cancer	1 (0.5)	2-yearRFS, %	91.5
Histologic type, n (%)		5-year RFS, %	63.9
Enteric features	47 (23.3)	5-year RFS for Mayo stage ≥III, %	28.4
Signet ring cell features	21 (10.4)	10-year RFS, %	59.8

LNs, lymph nodes; EGFR, epidermal growth factor receptor; CK-7, cytokeratin-7; CK-20, cytokeratin-20; OS, overall survival; CSS, cancer-specific survival; RFS, recurrence-free survival.

^Ŧ^KRAS overexpressioin was detected by immunohistochemical staining.

### Relapse, Survival and Prognostic Factor Analysis

**Figure 1:**
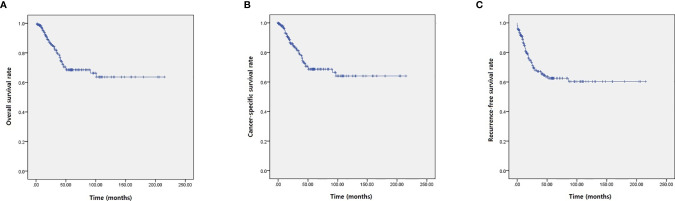
Kaplan–Meier analyses presenting survival outcomes of UC patients. **(A)** OS. **(B)** CSS. **(C)** RFS.

**Figure 2:**
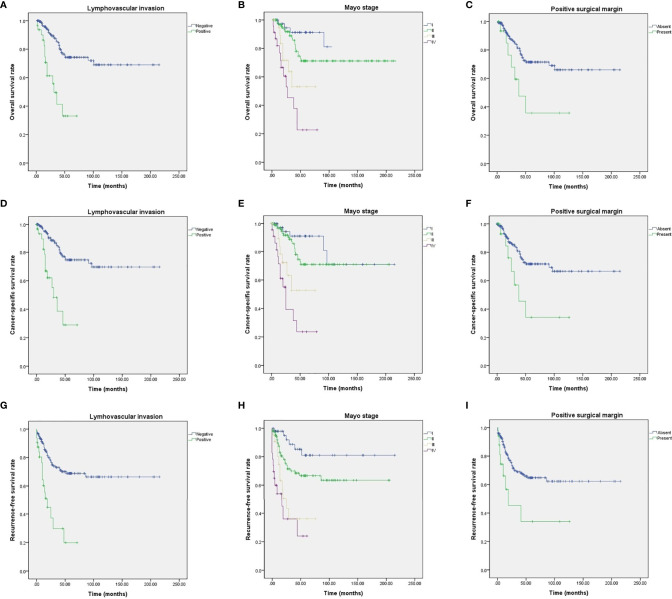
Kaplan–Meier analyses of UC patients with different survival outcome predictors. **(A)** OS for PLM. **(B)** OS for Mayo stages. **(C)** OS for PSM. **(D)** CSS for PLM. **(E)** CSS for Mayo stages. **(F)** CSS for PSM. **(G)** RFS for PLM. **(H)** RFS for Mayo stages. **(I)** RFS for PSM.

Among the study cohort, 82 patients (40.4%) had recurrence of urachal cancer after initial treatment (**Table 2**). For salvage treatment to the recurred UC patients, concurrent chemotherapy with radiotherapy (42 patients, 51.2%) was the most commonly used therapeutic modality (**Table 2**). Mean survival of OS, CSS, and RFS were 46.8, 44.7, and 39.6 months, respectively (**Table 2**, **Figure 1**). For OS, 2-, 5-, and 10-year survival rates were 98.0, 88.3, and 69.5%, respectively (**Table 2**, **Figure 1A**). 2-, 5- and 10-year CSS rates were 95.9, 83.1, and 68.4% (**Table 2**, **Figure 1B**), and RFS rates were 91.5, 63.9, and 59.8%, respectively (**Table 2**, **Figure 1C**). 5-year OS, CSS, and RFS for the patients with Mayo stage ≥III were 38.0, 35.2, and 28.4%, which indicated significantly poorer survival outcomes associated with advanced stage UC (**Table 2**, **Figure 2**). The results for survival predictors evaluated by multivariate Cox proportional hazards regression analyses are presented in **Table 3**. PSM and PLM were the independent predictors for shorter OS, CSS, and RFS (**Table 3**). Mayo stage ≥III and Sheldon stage ≥IIIC were also significantly associated with shorter survival outcomes including OS, CSS, and RFS (**Table 3**). Among pathologic types of UC, small cell cancer was an independent predictor for shorter OS and CSS (**Table 3**). However, body mass index (BMI) was associated with longer OS and CSS (OS: p = 0.037. CSS: p = 0.021) (**Table 3**). Regarding surgical methods, radical cystectomy was not associated with superior survival outcomes compared with partial cystectomy (**Table 3**).

**Table 3:** Predictors of survival rates.

Parameters	Overall survival			Cancer-specific survival			Recurrence-free survival		
	HR	95% CI	p-value	HR	95% CI	p-value	HR	95% CI	p-value
Mass excision method									
Partial cystectomy		Reference			Reference			Reference	
Radical cystectomy	1.420	0.631–3.193	0.397	1.392	0.619**-**3.129	0.424	1.619	0.816–3.209	0.168
TUR-B + partial cystectomy	0.849	0.204–3.530	0.822	0.843	0.203–3.503	0.814	0.980	0.305–3.152	0.973
Umbilectomy									
Not performed		Reference			Reference			Reference	
Performed	2.491	0.980–6.334	0.055	2.601	1.024–6.608	**0.044**	2.140	0.918–4.990	0.078
PLND									
Not performed		Reference			Reference			Reference	
Performed	1.253	0.646–2.428	0.505	1.264	0.652–2.450	0.488	1.589	0.911–2.770	0.102
BMI	0.911	0.835–0.994	**0.037**	0.912	0.837–0.995	**0.021**	0.987	0.918–1.062	0.727
DM	1.303	0.627–2.708	0.479	1.365	0.657–2.837	0.404	0.985	0.484–2.004	0.967
HTN	1.465	0.779–2.755	0.236	1.448	0.770–2.723	0.251	2.086	1.230–3.537	**0.006**
Age									
<54 years		Reference			Reference			Reference	
≥54 years	0.012	0.005–129.087	0.988	4.265	0.575–31.633	0.156	0.175	0.095–0.907	**0.041**
Mayo stage									
I: Tumor confined to urachus and/or bladder		Reference			Reference			Reference	
II: Tumor extending beyond the muscular layer of urachus and/or the bladder	1.885	0.707–5.025	0.205	1.871	0.702–4.987	0.211	2.423	1.005–5.839	**0.049**
III: Tumor infiltrating the regionallymphnode	5.556	1.737–17.772	**0.004**	5.483	1.215–19.535	**0.011**	6.012	2.212–16.341	**<0.001**
IV: Tumor infiltrating non-regional lymphnodes or other distant sites	10.559	3.716–30.001	**<0.001**	11.111	3.904–31.627	**0.001**	10.661	4.020–28.278	**<0.001**
Sheldon pathological staging system									
I: Confined to urachal mucosa		Reference			Reference			Reference	
II: Invasion confined to urachus	2.049	0.449–9.356	0.354	2.066	0.453–9.432	0.349	2.867	0.651–12.620	0.164
IIIA: To bladder	1.696	0.396–7.266	0.477	1.734	0.405–7.430	0.458	2.070	0.489–8.767	0.323
IIIB: Peri-urachal vesical fat	0.673	0.112–4.030	0.664	0.675	0.113–4.043	0.667	2.345	0.519–10.593	0.268
IIIC: To peritoneum	4.388	1.035–4.280	**0.039**	1.386	1.035–4.262	**0.017**	1.067	1.178–6.393	**0.043**
IIID: To viscera other than bladder	5.853	1.821–41.735	**0.018**	5.655	0.793–40.329	**0.039**	2.727	1.247–30.146	**0.013**
IV: Metastasis to other organs	7.891	1.262–49.319	**0.027**	7.812	1.254–48.669	**0.022**	6.109	1.006–37.113	**0.049**
Adjuvant chemotherapy									
None		Reference			Reference			Reference	
Adjuvant chemotherapy	0.010	0.003–99.288	0.984	0.128	0.003-83.217	0.983	0.104	0.001-1.052	0.972
Positive surgical margin									
Negative		Reference			Reference			Reference	
Positive	2.665	1.188–5.982	**0.017**	2.719	1.212–6.099	**0.015**	2.580	1.221-5.455	**0.045**
Positive lymphovascular invasion									
Negative		Reference			Reference			Reference	
Positive	4.561	2.379–8.743	**<0.001**	4.829	2.501–9.324	**<0.001**	3.853	2.163-6.863	**<0.001**
Pathologic type									
Adenocarcinoma		Reference			Reference			Reference	
Urothelical cancer	0.435	0.105–1.801	0.251	0.432	0.104–1.790	0.247	0.315	0.077–1.293	0.315
Undifferentiated cancer	1.550	0.213–11.298	0.665	1.500	0.206–10.931	0.689	0.012	0.005–7.999	**<0.001**
Small cell cancer	8.948	1.188–67.393	**0.033**	14.743	1.899–114.430	**0.010**	5.766	0.783–42.479	0.086
Histologic type									
Enteric features		Reference			Reference			Reference	
Signet ring cell features	2.556	0.740–83834	0.138	2.418	0.700–8.356	0.163	2.607	1.057–6.428	**0.037**
Squamous cell features	0.025	0.014–1.372	0.973	0.023	0.016–1.753	0.972	0.560	0.072–4.379	0.581
Transitional cell/adenomatous features	2.731	0.732–10.182	0.135	2.843	0.762–10.603	0.120	2.935	1.116–7.719	**0.029**
Mucinous features	2.307	0.897–5.936	0.083	2.228	0.866–5.733	0.097	1.201	0.590–2.444	0.613
Non-mucinous features	0.028	0.019–1.207	0.996	0.034	0.017–1.610	0.996	0.015	0.010–2.893	0.975

TUR-B, transurethral resection of bladder; PLND, pelvic lymphnode dissection; BMI, body mass index; DM, diabetes mellitus; HTN, hypertension.

The bold values indicate statistically significant P-values.

## Discussion

The current study retrospectively reviewed 203 patients treated for confirmed UC, and this study described their several unique and original clinicopathological findings. Moreover, this study also evaluated the clinicopathological predictors of oncological outcomes for UC. The mean age of our study cohort was 54.2 years, and 61.6% of the patients were male. These values are similar to the result of the recent SEER database analysis (age range 46–71 years/60% males) (13). Previous studies including Molina et al. have suggested strong association between Tobacco exposure and UC (14), and our study results also showed that more than half of the cohort (125 patients, 61.6%) were current or ex-smoker. As previous literatures demonstrated (2, 15), gross hematuria was the most common initial symptom. Solid mass lesion was the most commonly observed initial CT findings, and it provided a general impression of UC such as the size and location, which were consistent with previous reports (1, 16). Our study data presented that elevations in some tumor markers including CEA, and CA19-9 were accompanied in majority of the patients. Some previous researches including Siefker-Radtke et al. (17) showed similar results, and these tumor marker analyses strongly suggested diagnostic value of CEA and CA19-9 in UC although the disease-specificity for diagnosis might be relatively low. In addition, CEA or CA19-9 showed no association with patients’ survival in this study.

The currently accepted surgical treatment of UC throughout previous studies is partial cystectomy with complete resection of the tumor and *en-bloc* resection of the median umbilical ligament and the umbilicus is also recommened (1, 17, 18). However, this standard surgical modality and regional pelvic lymph node dissection (PLND) approach still carry some contraversies (18). In this study, most of the patients underwent partial cystectomy (82.8%), whereas 12 of them received TUR-B prior to partial cystectomy. Due to the retrospective multi-center based nature of this study, diversity of surgical modalities was inevitable. Nevertheless, umbilectomy with umbilical ligament resection and PLND were performed to only 5.9 and 23.2% of the patients, respectively. We reckon this deflection of surgical method might be due to the surgeon’s reluctance of extensive surgical dissection when the therapeutic effect of the method is not fully confirmed. Although PLND was not associated with survival of UC cohort in this study, we still believe the importance of PLND should not be diminished at any time. Since preoperative radiologic exams cannot detect all lymph invasions, pelvic exploring should be performed even if no lymph node invasion was suspected in preoperative imaging. In addition, further evaluations with larger size cohort would be helpful to define the surgical extent of PLND. Most of the previous studies about chemotherapy regimen for urachal adenocarcinoma have analyzed combination chemotherapy. As urachal adenocarcinoma is pathologically similar to colorectal adenocarcinoma, mFOLFOX-6 (leucovorin, fluorouracil, oxaliplatin) regimens have shown effective therapeutic outcomes in previous studies (17). Early analysis on the combination regimen of 5-fluorouracil, leucovorin, gemcitabine, and cisplatin also showed promising oncological outcomes with 30–40% of radiographic therapeutic response rates, but long-term outcomes need to be further evaluated (19). In addition, previous literatures have shown that the most effective chemotherapy regimen might be the combination of 5-fluorouracil and cisplatin, which seems to produce better outcomes in terms of response rate compared with other cisplatin-based regeimens (19, 20). Our study results showed that 12 different chemotherapy regimens were used, and even the most commonly used regimen (cisplatin with 5-fluorouracil) were applied to only 22 patient. Thus, it was relatively difficult to evaluate the optimal chemotherapy regimen with superior outcomes in the current therapy.

Although some previous studies tried to evaluate the immunohistochemical characteristics of UC, no UC specific immunohistochemical analysis based biomarkers have been found. Previous studies, which performed immunohistochemical analyses for UC, suggested several biomarkers including Ki67 and p53 (21, 22). Ki67 is expressed in proliferating cells and highly elevated expression of Ki67 is observed in UC (15, 21). An accumulation of p53 protein indicates mutations in tumor suppressor gene TP53, and strong positivity of p53 accumulation has been described in previous studies (22). Our study results showed increase of Ki57 expression and p53 positivity, but immunoreactivity of both p53 and Ki67 were not associated with survival outcomes of the patients.

According to a study that analyzed the UC cohort from MD Anderson Cancer Center, bone, lung, and liver were the three most common metastatic sites (10). Our study results showed that lung, liver, and skin were the three most metastatic sites of metastasis when peritoneum and abdominal wall are excluded from the analysis. These study results emphasize the importance of regular evaluation of lung and liver for monitoring the progression and recurrence of UC.

Another noticeable finding of this study is the clinical significance of PSM and PLM on survival of UC patients. Many previous studies including Ashley et al. (23) presented PSM has a strong negative impact on survival of UC cohort. Our study results also showed that PSM is significantly associated with shorter OS, CSS, and RFS. In addition, PLM was also independent predictor of OS, CSS, and RFS in our study. To our knowledge, the current study is the first research presenting the influence of PLM on survival of UC cohort.

The mean values of OS, CSS, and RFS in this study are similar to previous studies (24). However, 5- and 10-year survival rates are relatively higher than previous studies (10, 14, 25). We believe these better long-term survival rates are mainly due to small cohort size and relatively longer follow-up period. In the current study, 25.1% of the patients, who had excellent oncological outcomes such as no UC recurrence, underwent surgery more than 10 years ago. Thus, longer follow-up period of these patients might have exaggerated survival rates of the entire study cohort. Moreover, overall 203 patients are included in this study, which implies the cohort size was not big enough to minimize the selection bias affecting patients’ long-term survival analysis.

Although no confirmative staging system for UC has been validated, Sheldon and Mayo staging systems are the two most commonly used stages (23, 25, 26). The current study results showed that higher tumor stages (Mayo stage ≥III and Sheldon stage ≥IIIC) were strongly associated with poor survival outcomes of UC patients, and these results coincide with the previous study results (20, 25). As higher tumor stages of UC significantly increased negative prognostic predictive ability of the Mayo and Sheldon staging systems, early and active therapeutic intervention might need to be emphasized for the patients having UC with progressed stages.

There are some potential limitations in this study. First, due to the retrospective multi-center based nature of the study, standardization of therapeutic modalities was not performed. This limited evaluation of therapeutic methods on survival outcomes. Second, because of the rareness of UC, relatively small sample size diminished statistical power of the study results including immunohistochemical biomarkers. Thus, further research studies with larger cohort size need to be undertaken to confirm the study results.

Despite the limitations, to the best of our knowledge, this study is the first multi-institutional study with the largest sample size, which evaluated therapeutic outcomes and potential predictors of UC patients in Asia.

## Conclusions

Immunohistochemical biomarkers including Ki67 and p53 are markedly increased in UC patients. The strong association of PSM, PLM with survival outcomes of UC patients emphasizes an importance of the complete surgical resection of tumor lesion. Higher Mayo and Sheldon stages were significantly associated with long-term survival. Due to the relatively small cohort size, the universal predictors of oncological outcomes in UC patients were not confirmed.

## Data Availability Statement

The original contributions presented in the study are included in the article/supplementary material. Further inquiries can be directed to the corresponding author.

## Ethics Statement

The studies involving human participants were reviewed and approved by Seoul National University Bundang Hospital IRB. The patients/participants provided their written informed consent to participate in this study.

## Author Contributions

YDY: conceived of the presented idea, analysis and interpretation of data, had full access to all the data in the study and takes responsibility for the integrity of the data and the accuracy of the data analysis, writing—initial draft, critical revision of the article for important intellectual content, statistical analysis, and administrative, technical, and administrative support. JO: study concept and design, acquisition of data, analysis and interpretation of data, had full access to all the data in the study and takes responsibility for the integrity of the data and the accuracy of the data analysis, editing—initial draft, critical revision of the article for important intellectual content, statistical analysis, administrative, technical, and material support, and study supervision. YK, JWK, SJ, SK, JP, HS, HK, BJ, T-HK, SC, JN, JYK, KJ, WJ, YEY, SY, and S-HH: study concept and design, acquisition of data, and administrative, technical, and material support. All authors contributed to the article and approved the submitted version.

## Funding

This research was neither funded nor supported by any institution or organization. The authors wish to acknowledge all participating investigators.

## Conflict of Interest

The authors declare that the research was conducted in the absence of any commercial or financial relationships that could be construed as a potential conflict of interest.
